# Purification and Characterization of a Novel and Robust L-Asparaginase Having Low-Glutaminase Activity from *Bacillus licheniformis: In Vitro* Evaluation of Anti-Cancerous Properties

**DOI:** 10.1371/journal.pone.0099037

**Published:** 2014-06-06

**Authors:** Richi V. Mahajan, Vinod Kumar, Vinoth Rajendran, Saurabh Saran, Prahlad C. Ghosh, Rajendra Kumar Saxena

**Affiliations:** 1 Department of Microbiology, University of Delhi South Campus, New Delhi, Delhi, India; 2 Department of Biochemistry, University of Delhi South Campus, New Delhi, Delhi, India; 3 Technology Based Incubator, University of Delhi South Campus, New Delhi, Delhi, India; Boston University Goldman School of Dental Medicine, United States of America

## Abstract

L-asparaginase having low glutaminase has been a key therapeutic agent in the treatment of acute lymphpoblastic leukemia (A.L.L). In the present study, an extracellular L-asparaginase with low glutaminase activity, produced by *Bacillus licheniformis* was purified to homogeneity. Protein was found to be a homotetramer of 134.8 KDa with monomeric size of 33.7 KDa and very specific for its natural substrate *i.e.* L-asparagine. The activity of purified L-asparaginase enhanced in presence of cations including Na^+^ and K^+^, whereas it was moderately inhibited in the presence of divalent cations and thiol group blocking reagents. The purified enzyme was maximally active over the range of pH 6.0 to 10.0 and temperature of 40°C and enzyme was stable maximum at pH 9.0 and −20°C. CD spectra of L-asparaginase predicted the enzyme to consist of 63.05% α- helix and 3.29% β-sheets in its native form with T_222_ of 58°C. Fluorescent spectroscopy showed the protein to be stable even in the presence of more than 3 M GdHCl. Kinetic parameters K_m_, V_max_ and k_cat_ of purified enzyme were found as 1.4×10^−5^ M, 4.03 IU and 2.68×10^3 ^s^−1^, respectively. The purified L-asparaginase had cytotoxic activity against various cancerous cell lines *viz.* Jurkat clone E6-1, MCF-7 and K-562 with IC_50_ of 0.22 IU, 0.78 IU and 0.153 IU respectively. However the enzyme had no toxic effect on human erythrocytes and CHO cell lines hence should be considered potential candidate for further pharmaceutical use as an anticancer drug.

## Introduction

The inability of leukemic cells to synthesize their own L-asparagine has made the enzyme L-asparaginase a key component of chemotherapy in the treatment of acute lymphoblastic leukemia (A.L.L) [1], [2]. This enzyme hydrolyses L-asparagine into L-aspartic acid and ammonia in blood vascular system, making the leukemic cells devoid of essential exogenous L-asparagine, leading to inhibition of protein synthesis and apoptosis [3]–[5], hence making this enzyme a potent anti-cancerous agent.

The current commercial supply of L-asparaginase is chiefly derived from *E. coli* and *Erwinia chrysanthemi* but the drug from these sources are strongly immunogenic hence neutralizing the therapeutic effects and causing symptoms in more than 50% of cancer cases [6]. Also, many studies have observed the increase in number of patients with clinical resistance to the current market drug [6]–[8]. The commercial L-asparaginase was found to have *in vitro* resistance against the cells from patients with relapsed A.L.L [9]. Another problem associated with the commercial L-asparaginases is its low substrate specificity and high glutaminase activity, which can cause liver dysfunction, pancreatitis, leucopenia, neurological seizures, and coagulation abnormalities leading to intracranial thrombosis or hemorrhage [10]. Hence there is a need for novel and robust L-asparaginases from GRAS (*Generally Recognized as Safe*) microorganisms which has improved stability, lower glutaminase activity with high substrate affinity, low K_m_ value and sufficient half-life under physiological conditions so that it can overcome the above mentioned challenges faced in the current scenario.

Therefore, the present study is targeted to purify L-asparaginase from *Bacillus licheniformis* RAM-8 (soil Isolate) which has been confirmed to be a novel L-asparaginase and has been evaluated for its biophysical and biochemical characteristics and its potential as an anticancer agent against different human cancer cell lines.

## Materials and Methods

*Ethics statement:* The blood samples in the current work were obtained from Rotary Blood Blank, New Delhi as a gift and these blood samples are commercially available in the Rotary Blood Bank for research purposes. Ethical committee approval is not necessary for obtaining human blood for research purposes. We have been using human blood for research purposes obtained from Rotary Blood Bank and published the same previously [11].

### 2.1 Chemicals

Chemicals used for enzyme purification (chromatographic matrices) and characterization were purchased from Sigma Aldrich. Nessler’s reagent was purchased from Fluka (Buchs, Switzerland). L-asparagine was purchased from Spectrochem (India). All chemicals used for production were of analytical grade and were purchased from Hi-Media (India). Chemicals and markers used in native and SDS PAGE were obtained from BioRad.

### 2.2 L-asparaginase Production

L-asparaginase production by *Bacillus licheniformis* RAM-8 (soil isolate; identified on the basis of 16 s RNA and 500 bp analysis; Midi-labs, USA) was carried out in optimized modified Czapek Dox medium [12]: Na_2_HPO_4_, 6 g/L; KH_2_PO_4_, 2 g/L; NaCl, 0.5 g/L; L-asparagine, 20 g/L; glycerol, 2 g/L; MgSO_4_.7H_2_0, 0.2 g/L; CaCl_2_.2H_2_O, 0.005 g/L. Flasks containing production medium (50 ml in 250 ml flask) were inoculated with 2% inoculum (v/v) (O.D. 2.0). Incubation was carried out at 37°C at 200 rpm for 24 h. The enzyme production was extracellular and the enzyme activity was determined using the culture filtrate. All the experiments were carried out in triplicate and the mean values with standard deviations (SD) were calculated.

### 2.3 Enzyme Assay

Assay of enzyme was carried out as per the nesslerization procedure given by Shirfrin *et al*. [13], using 189 mM L-asparagine as substrate or mentioned otherwise, in 50 mM Tris-HCl buffer (pH 8.6) and reading the absorbance at 436 nm. Ammonium sulfate solution was used for preparation of standard curve. One international unit (IU) of asparaginase activity is defined as the amount of enzyme required to release one µmole of ammonia per ml per minute at pH 8.6 at 37°C.

### 2.4 Enzyme Purification

#### 2.4.1 Ultrafiltration

The cell free fermentation broth was subjected to ultrafiltration with membrane cartridge of 30 kDa to remove the lower proteins and to concentrate the desired protein. Retentate and permeate collected after ultrafiltration were analyzed for L-asparaginase activity and protein concentration using Bradford method [14].

#### 2.4.2 Acetone precipitation

Chilled acetone (−20°C) was added to the concentrated broth with constant stirring at 4°C in the gradient concentration of 20 to 80%, for proteins to precipitate. The fractions showing maximum activity were centrifuged air dried and dissolved in minimal amount of 50 mM Tris HCl (pH 8.6) and dialyzed against the same buffer to obtain the concentrated protein.

#### 2.4.3 DEAE cellulose chromatography

The concentrated enzyme was applied to diethylaminoethyl cellulose column (DEAE cellulose) (4×60 cm) equilibrated with 50 mM Tris-HCL (pH-8.6). The column was washed with 2 volumes of starting buffer and the protein was eluted with linear gradient of NaCl (0–0.5 M) prepared in phosphate buffer pH-7.4 at the rate of 60 ml per hour. Fractions showing L-asparaginase activity were pooled together dialyzed against 50 mM Tris-HCL (pH-8.6) and concentrated with bench top protein concentrator at 4°C.

#### 2.4.4 Gel filtration

The concentrated enzyme solution was added on the top of sephadex G-100 column (4×60) cm equilibrated with 50 mM Tris-HCl (pH-8.6) and eluted with the same buffer at the flow rate of 0.5 ml per minute. Fractions showing L-asparaginase activity were pooled and dialyzed against the same buffer and lyophilized with bench top lyophilizer. The homogeneity of the protein was checked by SDS and native PAGE.

### 2.5 Characterization of Purified L-asparaginase

#### 2.5.1 Determination of molecular weight

The purified L-asparaginase was applied on to Sephacryl™ S-200 high resolution column (Pharmacia, 16/60) equilibrated with 50 mM tris-HCl (pH-8.6) and eluted at flow rate of 0.5 ml per minute [15]. The standard molecular marker proteins were applied to the column and the elution volume (V_e_) of each marker protein and void volume (V_o_) of the column were recorded. The molecular mass of the protein was determined on a semi-log graph by plotting V_e/_V_o_ on X-axis and logarithm of molecular weight on Y-axis.

#### 2.5.2 Effect of pH and temperature on the activity and stability of enzyme

The activity of L-asparaginase was evaluated at different pH values and temperature. Optimum pH for L-asparaginase activity was determined over a pH range of 4 to 10. For pH stability studies, the enzyme preparations were incubated at pH of 4–10 for 24 h at 4°C and residual activity was determined under assay conditions (pH 8.6, 50 mM). The optimum temperature range for enzyme activity was determined by carrying out the enzyme assay at different temperatures ranging from 25 to 65°C. Also the heat stability of enzyme was determined by incubating the lyophilized enzyme at different temperatures *viz* −20°C, 4°C, 10°C, 30°C and 60°C for 30 days.

#### 2.5.3 Substrate specificity

Enzyme activities were evaluated with different amides as substrates, *viz.* L-asparagine, D-asparagine, L-glutamine, D-glutamine, L-aspartic acid, D-aspartic acid, L-glutamic acid, L-ornithine and urea, at concentrations of 10 mM, respectively. Results were presented in the terms of percentage relative activity.

#### 2.5.4 Effect of various metal ions, serum components and inhibitors on enzyme activity

Effect of different inorganic ions on enzyme activity was determined by pre-incubating the enzyme with different salts at the concentration of 100 mM for 6 hours at 4°C. Also the effect of serum, serum components and inhibitors (sulfhydryl and serine) was determined by incubating the enzyme at the effector concentration of 10 mM for 6 hours at 4°C. Results were presented in the terms of percentage relative activity.

#### 2.5.5 Kinetic parameters

The *V*_max_, *K_m_*, *k*_cat_, and *k*_cat_/*K_m_* were determined at 25°C using L-asparagine as substrate at the concentrations ranging from 2 to 20 mM with the constant enzyme concentration of 1 mg/ml. The initial turnover rate at ten different concentrations of substrate was calculated, and the type of inhibition and Michaelis-Menten parameters were determined from Lineaweaver-Burk plots by using the equation derived from the linear-regression analysis of curve. The *k*_cat_ value was calculated by applying the equation *k*_cat_ = *V*_max_/[E], where [E] is the enzyme concentration in the assay. k_cat_ and specificity constants (k_cat_/K_m_) were calculated on the basis of one active site per 33.7 kD subunit as described by Kumar *et al*. [16].

#### 2.5.6 Circular dichroism

CD spectra were recorded on a JASCO J-815 spectropolarimeter (JASCO Corporation, Hachioji-shi, Tokyo, Japan) using a cylindrical quartz cell of path length 1 mm and 1 cm, respectively, for far and near- UV region. Changes in the secondary and tertiary structure of the protein were monitored in the far and near -UV region between 190–260 nm and 260–320 nm, respectively. Three consecutive spectral scans were averaged and corrected by subtracting corresponding blanks and subjected to noise reduction. The unfolding transition pattern of purified L-asparaginase was monitored by far UV CD spectroscopy, where changes in signal intensity at 222 nm were plotted against the function of temperature.

#### 2.5.7 Fluorescence spectroscopy

Fluorescence measurements were carried out using Eclipse Cary Varian UV-Vis spectrofluorometer (Varian, Inc. Hansen Way, Palo Alto, CA, USA) attached to a Peltier temperature controller. An excitation wavelength of 280 nm with an excitation slit of 5 nm and an emission slit of 5 nm was used and the fluorescence was recorded from 300 nm to 400 nm. Unfolding pattern and stability of protein was determined using 0–6 M of guanidine HCl (GdHCl) as described by Bansal *et al.*, [17]. The fluorescence intensity was plotted as the function of wavelength using F_400_ nm as the baseline.

#### 2.5.8 N-terminal sequencing of L-asparaginase

In order to confirm the identity of the protein, protein samples corresponding to a molecular mass of 33.7 kDa on SDS-PAGE were blotted onto a polyvinyl difluoride (PVDF) membrane (Sigma-Aldrich, USA) and subjected to N-terminal amino acid sequence determination using an automated protein sequencer PPSQ31A (Shimadzu, Japan).

### 2.6 Anti-tumor Application of L-asparaginase from *Bacillus licheniformis*

#### 2.6.1 Cells and cell culture conditions

The anticancer properties of purified L-asparaginase were evaluated on different human tumor cell lines *viz.* Jurkat clone E6-1, MCF-7 and K-562 (procured from NCCS, Pune). The Jurkat cells and K-562 were grown as suspension in RPMI-1640 medium and MCF-7 were grown in DMEM medium supplemented with 10% heat inactivated FBS (Sigma), penicillin (100 µg/ml) and streptomycin (100 µg/ml). Cells were maintained under a fully humidified atmosphere of 95% room air and 5% CO_2_ at 37°C.

#### 2.6.2 Cell viability measurement

The anti-proliferative effect of L-asparaginase was measured by the colorimetric assay that measures the reduction of yellow 3-(4,5-dimethythiazol-2-yl)-2,5-diphenyl tetrazolium bromide (MTT) by mitochondrial succinate dehydrogenase to an insoluble, colored (dark purple) formazan product which is solubilized in organic solvent and measured spectrophotometrically. Briefly, cells were plated at a cell density of 1×10^4^ cells/well on flat bottomed 96-Well standard microplates. The purified L-asparginase (100 IU/mg) was serially diluted in incomplete medium and added to cell cultures at a final concentration from 0.75–25 µg/ml. After incubation for 24 hr, 20 µl of MTT at a concentration of 5 mg/ml in phosphate-buffer saline (PBS, pH 7.4) were added to each well. After 4 h of incubation the plates were centrifuged at 2000 rpm for 10 min, after which plates were rapidly inverted with a firm flick to remove the culture medium. To solubilize the formazan precipitates by the addition of 150 µl of 1∶1 mix of ethanol and DMSO were added to each well and the plates were further incubated for 20 min at room temperature. The absorbance was then determined by spectrophotometric microplate reader Tecan infinite 200 pro, at a test wavelength of 550 nm and a reference wavelength of 620 nm.

#### 2.6.3 *In vitro* hemolysis test

The purified L-asparaginase was tested for in vitro hemolysis by incubating the increasing concentrations of the enzyme (6.25 µg to 100 µg) with heparinized human whole blood (obtained from Rotary Blood Bank, New Delhi) in phosphate buffer (pH 7.2) for 24 h as described by Lubran [18]. The reaction was terminated by adding 2.5% gluteraldehyde after 24 h of incubation and then centrifuged. The supernatant was read at 540 nm against negative control i.e. sample without enzyme. The preparation of human blood in double distilled water was treated as positive control.

## Results

### 3.1 Purification of L-asparaginase and Molecular Mass Determination

**Figure 1:**
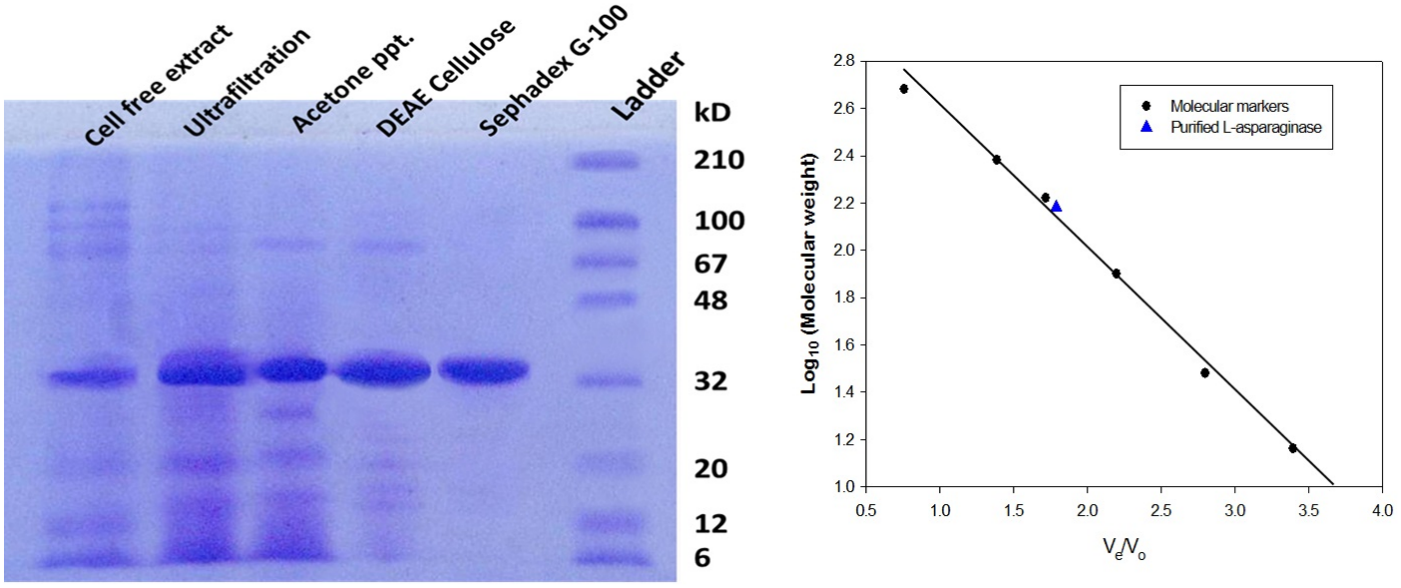
Molecular weight determination of purified enzyme (a) SDS PAGE for steps of purifying L-asparaginase; b) Plot of V_e_/V_o_ against semi-log of molecular weight of proteins on Sephacryl TM S-200 high resolution column (16/60) for α-Lactalbumin (12.4 kDa), carbonic anhydrase (30 kDa), bovine serum albumin (66 kDa), yeast alcohol dehydrogenase (150 kDa), sweet potato β-amylase (200 kDa) and ferritin (450 kDa).

The stepwise purification of L-asparaginase from *Bacillus licheniformis* RAM-8 has been presented in Table 1 and Figure 1a. The series of chromatography steps were very effective and gave overall purification of 33-fold. The final protein content of the purified L-asparaginase was 15.25 mg, with the yield of 32.95%. The specific activity of purified enzyme was 697.09 IU/mg of protein. Protein was found to have molecular size of 134.8 kDa (Figure 1b) as determined through gel-filtration chromatography and monomeric size of 33.7 kDa confirmed by SDS-PAGE. The theoretical IEF of enzyme was calculated to be 5.48.

**Table 1:** Stepwise purification of L-asparaginase from *Bacillus licheniformis.*

Purification steps	Total enzyme units	Total protein (mg)	Specific activity (IU/mg)	Yield (%)	Purification fold
Culture filtrate	32,260	1396	23.10	100	1.00
Ultra filtration	29,587	862	30.84	94.81	1.13
Acetone precipitation (80%)	24,392	459	42.77	82.06	1.85
Anion exchange (DEAE cellulose)	15,898	37.90	419.14	49.28	18.09
Gel filtration (Sephadex G-150)	10,632	15.25	697.09	32.95	30.17

### 3.2 Effect of pH and Temperature on Activity and Stability of L-asparaginase

**Figure 2:**
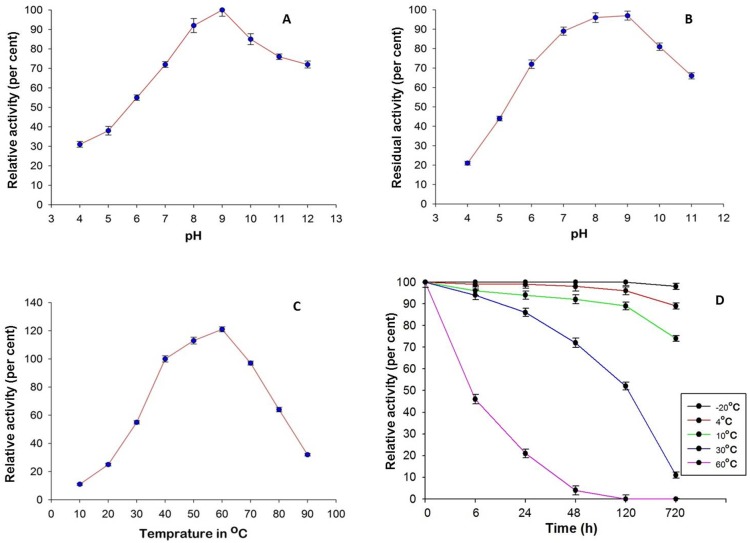
Effect of physical parameters on purified L-asparaginase a) Effect of pH on enzyme activity; b) Effect of pH on the stability of enzyme; c) Effect of temperature on assay reaction; d) Heat stability of enzyme. 100% activity corresponds to 140 U of enzyme. Error bars represent SD of three experiments.

Purified L-asparaginase from *Bacillus licheniformis* RAM-8 was active over wide range of pH of 6–11 with maximum activity at pH 9 (Figure 2a). It is evident from Figure 2c that the purified enzyme was active at the temperature range of 30–50°C and showed a steep decent in activity above 60°C. Also the enzyme was maximally stable at pH range of 7.0 to 9.0 over period of 24 hours (Figure 2b). The best temperature for enzyme storage was found to be −20°C where it was even stable after 30 days of keeping (Figure 2d).

### 3.3 Effect of Metal Ions, Serum Components and Inhibitors on L-asparaginase Activity

**Figure 3:**
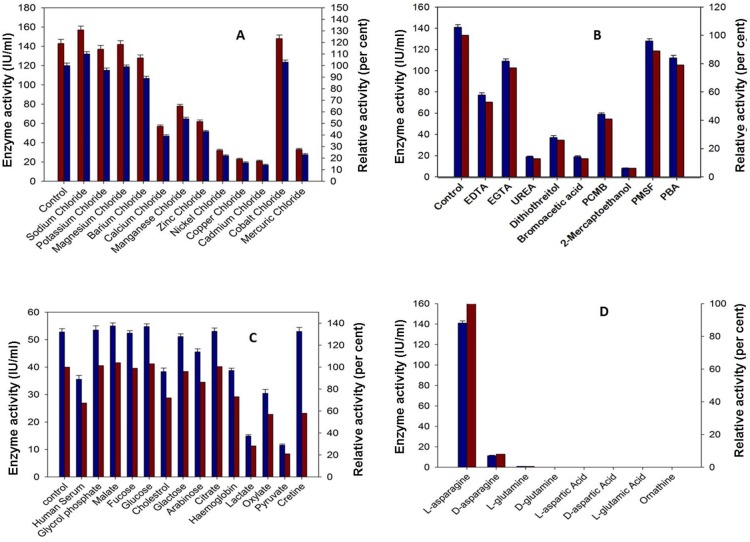
Effect of chemical parameters on purified L-asparaginase a) Effect of metal ions (100 mM) on enzyme activity; b) Effect of inhibitors (10 mM) on enzyme activity (EDTA: Ethylenediaminetetraacetic acid, EGTA: Ethylene glycol tetraacetic acid, PCMB: p-chloromercuribenzoic acid, PMSF: Phenylmethanesulfonylfluoride, PBA c) Effect of serum and serum components (10 mM) on enzyme activity; d) Substrate (10 mM) specificity of enzyme. 100% activity corresponds to 140 U of enzyme. Error bars represent SD of three experiments.

It is evident from the Figure 3a that enzyme activity enhanced considerably in the presence of most the monovalent cations and was maximal in the presence of Na^+^, K^+^ and Mg^++^. However, the other divalent cations had detrimental effect on the enzyme activity. The enzyme retained more than 80% activity in the presence of serine protease inhibitors viz. PMSF & PBA hence indicating that it is not a serine hydrolase. There was decrease of 60% in the activity of L-asparaginase from *Bacillus licheniformis* RAM-8 in the presence of sulfhydryl inhibitors. Also, dissociating agents urea and EDTA inhibited the activity by 70% (Figure 3b).

However purified L-asparaginase activity was robust at physiological pH and temperature but the enzyme activity decreased by approximately 40%, when the purified L-asparaginase was incubated with human serum and its components respectively under *in vitro* conditions for 48 hour wherein on detailed evaluation it was obtained that lactate, oxylate and pyruvate exhibited more than 70% inhibition (Figure 3c).

### 3.4 Substrate Specificity

The purified L-asparaginase showed a high specificity against its natural substrate i.e. L-asparagine. However, less than 10% and 1% activity against D-asparagine and L-glutamine, respectively was observed (Figure 3d). No other substrate was found to interact with the enzyme in its pure form.

### 3.5 Kinetic Parameters

**Figure 4:**
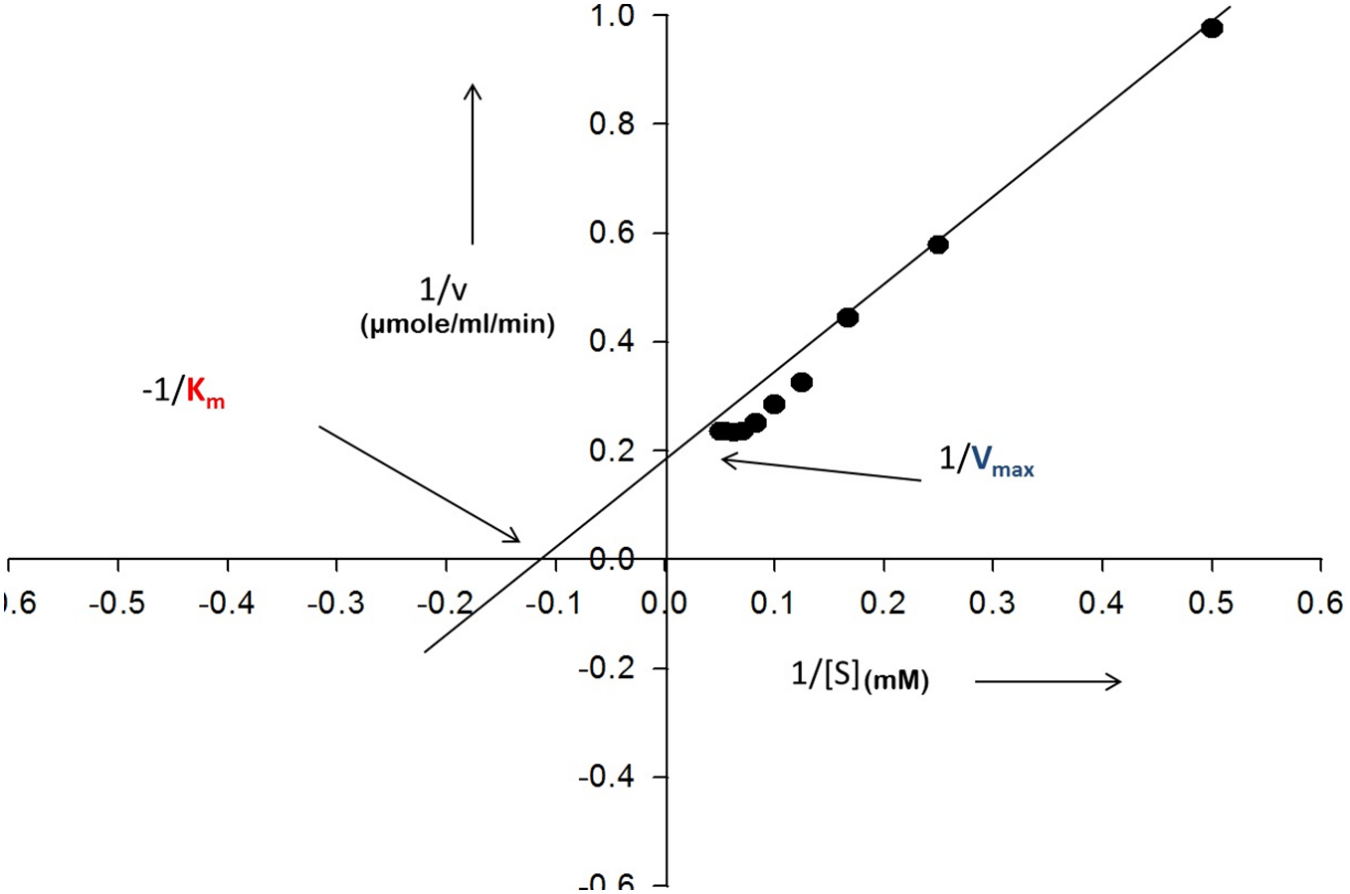
Lineweaver Burk plot for determining the kinetic parameters of purified L-asparaginase from *Bacillus licheniformis.* K_m_ = 1.4×10^−5^ M V_max_ = 4.03 IU/µg.

The Lineweaver Burk plot in Figure 4 infers that K_m_ and V_max_ of purified L-asparaginase from *Bacillus licheniformis* RAM 8 using L-asparagine as substrate was 1.4×10^−5^ M and 4.03 IU, respectively and turnover number (K_cat_) of enzyme was determined to be 2.68×10^3 ^s^−1^. (K_cat_/K_m_) of the enzyme was found to be 1.503×10^6^ M^−1^s^−1^.

### 3.6 Circular Dichroism

**Figure 5:**
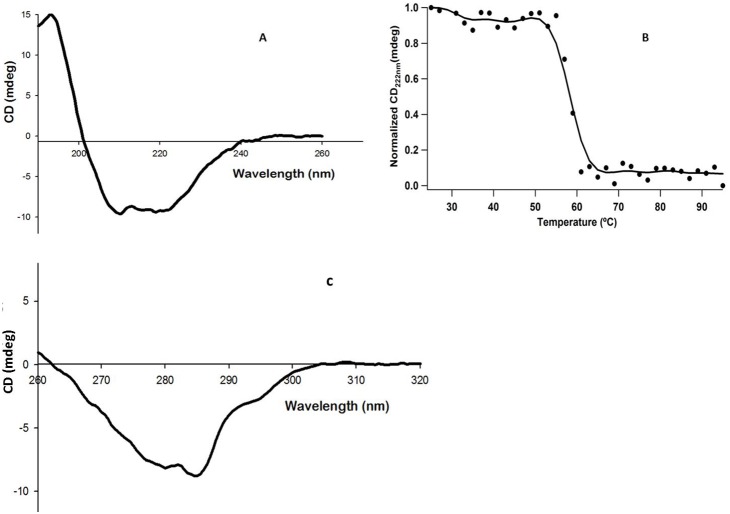
CD spectra of purified L-asparaginase a) Far UV CD spectra of L-asparaginase at 0.1 mg/ml in 0.1 M tris HCL (pH-8.4); b) Melting temperature of enzyme (T_222__nm_). c). Near UV CD spectra of purified L-asparaginase 1.0 mg/ml in 0.1 M Tris HCL (pH-8.4).

CD spectrum of purified L-asparaginase gave negative elipticities at 208 and 222 nm (Figure 5a). The k2D analysis of the far UV CD spectrum from 240 to 200 nm predicted apha helix to be 63.05% and beta sheets to be 3.29% and T_m_ of protein was found to be 58°C which state its reason for steep decent in the activity when enzyme was tested above 60°C (Figure 5b). The near UV CD spectra of the protein gave negative elipticities in the range of 260 to 290 as presented in Figure 5c.

### 3.7 Fluorescence Spectroscopy

**Figure 6:**
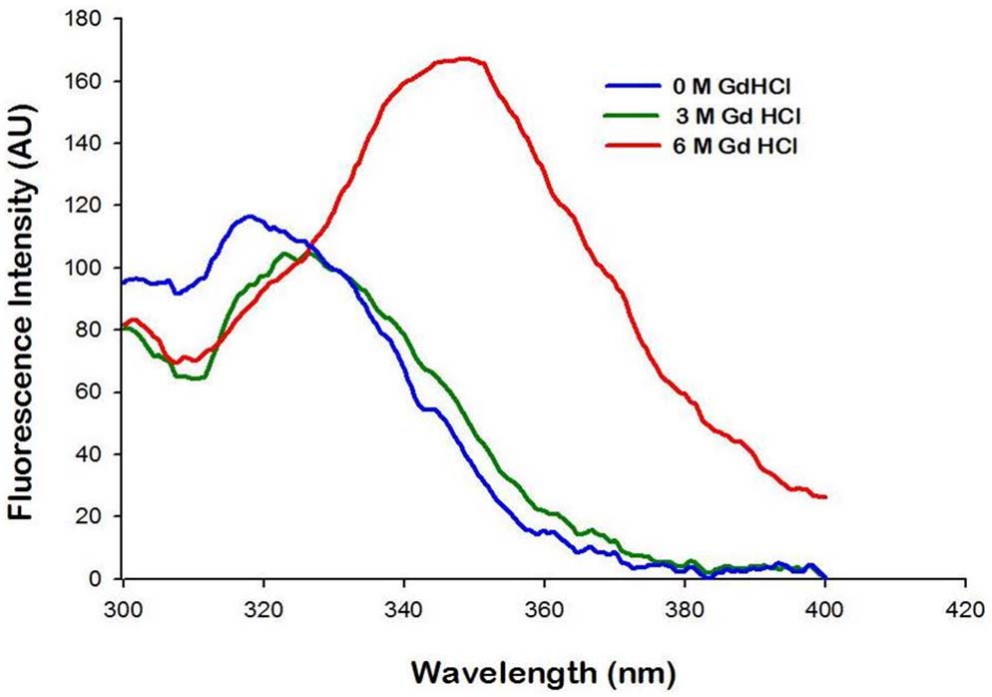
Fluorescence spectroscopy showing the emission spectrum in the range of 300–400 **nm (excitation wavelength: 292 nm) and unfolding transitions of L-asparaginase at 0** **M, 3** **M and 6** **M guanidine HCl.**

The purified L-asparaginase protein showed maximum fluorescence at 323 nm in its native form in the absence of GdHCl, however a shift to 352 nm was observed when 6 M GdnCl was added thus stating its complete unfolding as presented in Figure 6. A higher fluorescence was observed in the protein in its unfolded cold state.

### 3.8 N-terminal Sequencing of L-asparaginase

The protein samples corresponding to molecular mass of 33.7 kDa on SDS-PAGE were blotted on a polyvinyl difluoride (PVDF) membrane (Sigma-Aldrich, USA) and were subjected to the N-terminal amino acid sequence determination using an automated protein sequencer PPSQ21A (Shimadzu, Japan). The sequence of the first 15 N-terminal amino acid residues was found to be **D N K K V E A A T G G T Q A G** which showed a wide variation with the known and reported protein sequence of L-asparaginase.

### 3.8 Anti-proliferative Effect of L-asparaginase

**Figure 7:**
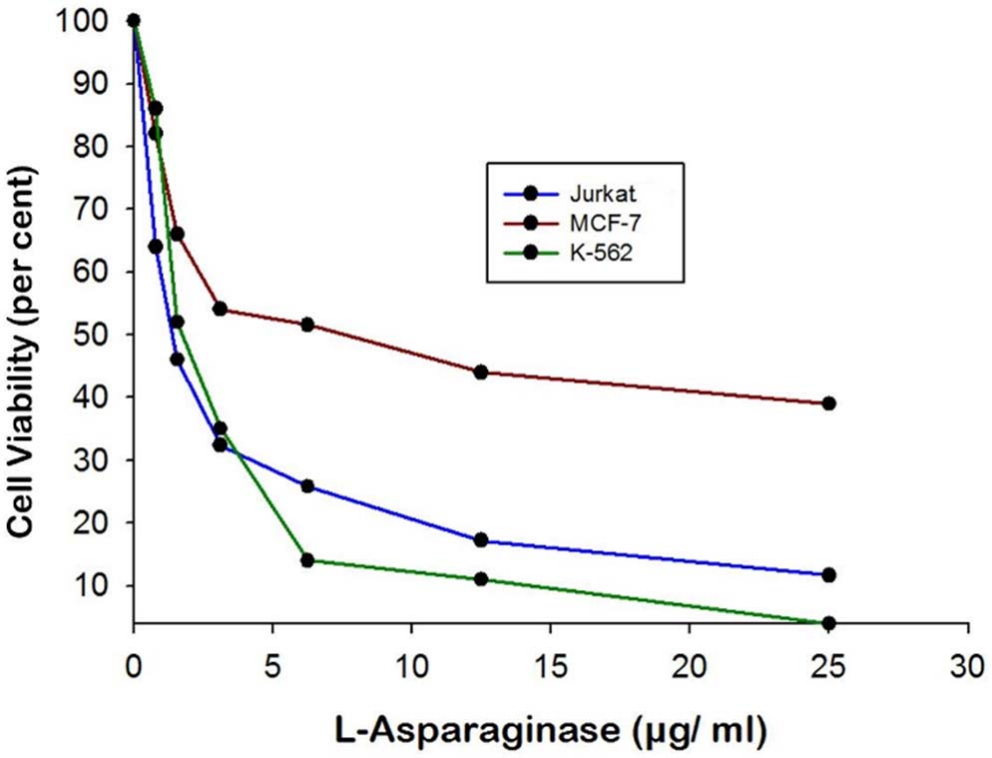
Anti-cancerous effect of enzyme on different human cancer cell lines *viz*. Jurkat clone E6-1, MCF-7 and K-562.

The anti-proliferative activity of L-asparaginase was monitored against three different human tumor cell lines viz. Jurkat clone E6-1, MCF-7 and K-562. The IC_50_ of purified L-asparaginase from *Bacillus licheniformis* RAM-8 was found to highly effective against the leukemic cell lines viz. Jurkat clone E6-1 cell lines and K-562 cell lines with IC_50_ of 0.22 IU and 0.15 IU, respectively (Figure 7). The breast cancer cell line MCF-7 also showed a retarded growth against the increasing concentrations and showed an IC_50_ of 0.78 IU.

### 3.9 *In vitro* Hemolysis Test for Testing the Drug Toxicity

**Figure 8:**
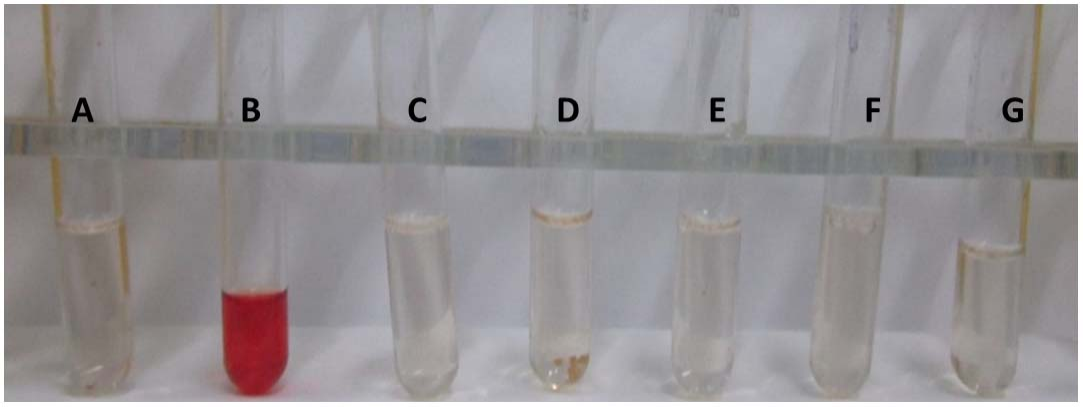
*In vitro* hemolysis test for testing the L-Asparaginase toxicity. A: negative control (without L-asparaginase); B: positive control (with double distilled water); C: 100 µg/ml; D: 50 µg/ml; E: 25 µg/ml; F: 12.5 µg/ml; G: 6.25 µg/ml of L-asparaginase.

The L-asparaginase incubated with the heparinized blood showed no effect even at concentration of 100 µg/ml (Figure 8). Also the drug did not have the inhibitory effect on the test cell line Chinese hamster ovary (CHO) cells hence the drug can be regarded safe for the further *in vivo* evaluations.

## Discussion

Purification of L-asparaginase from *Bacillus licheniformis* RAM-8 was achieved by using ultra-filtration, 80% acetone precipitation, DEAE cellulose chromatography and sephadex G-100 gel filtration, respectively. L-asparaginase from *Bacillus licheniformis* RAM-8 was purified to apparent homogeneity with the purification fold of 30.17 and a yield of 32.95% where the specific activity of enzyme increased from 23.10 IU/mg to 697.09 IU/mg. Protein had a molecular weight of 134.8 kDa but monomeric size of 33.7 kDa hence could be a possible homotetramer. These findings are in accordance with the previous reports stating that bacterial L-asparaginase is a homotetramer [19]–[21]. The theoretical IEF of enzyme was calculated to be 5.48, close to that of L-asparaginase of *E. coli* (IEF 5) but different from that of *Erwinia* (IEF 8.7) [16], [22]. Purified L-asparaginase from *Bacillus licheniformis* RAM-8 was functionally stable and active over wide range of pH and temperature, and showed the optimum activity under physiological conditions. Hence, this L-asparaginase was more robust as compared to L-asparaginase from *E. coli* and *Erwinia* [16],[22]–[23]. Most monovalent cations, Mg^++^ and serum sugars enhanced the enzyme activity, while enzyme activity decreased in the presence of other divalent cations and serum organic acids. In this respect, the enzyme was similar to *Erwinia* L-asparaginase [24]. The inhibitory effect of divalent cations such as Ca^++^ and serum organic acids may be overcome by the entrapment of the enzyme in liposomes which may mask the inhibitory effect. The enzyme entrapment may further prolong the plasma half-life and also reduce the immunogenicity, further enhancing the therapeutic efficiency as compared to free enzyme [25]. Activity of L-asparaginase decreased significantly by 60% in presence of sulfhydryl inhibitors, which may be due to presence of free –SH groups at the active sites of enzyme [26]. The enzyme was found to be very specific for its natural substrate L-asparagine, with less than 1% glutaminase activity. However the *E. coli* L-asparaginase is associated with significant glutaminase activity [2]. It is reported that deleterious effects are encountered when glutamine is depleted below critical levels, reducing synthesis of several important proteins *viz*. albumin, insulin, fibrinogen and protein-C [10], [25]. Lower glutaminase activity also enhances L-asparagine depletion [25]. The purified enzyme has K_m_ value of 1.4×10^−5^ M which is lower than L-asparaginase of *Erwinia* (3.0×10^−3^ M) [27] and *E. coli* (3.5×10^−3^ M) [28] and hence a better substrate affinity, though lower K_m_ value of 7.4×10^−6^ M was obtained in case of L-asparaginase from *Vibrio succinogenes* [28]. CD spectrum of the purified L-asparaginase showed negative elipticities at 208 and 222 nm which are characteristic of spectra obtained for mixed alpha/beta types of proteins [29]. The near UV CD spectra of the protein gave negative elipticities in the range of 260 to 290 which may be attributed to the presence of the aromatic amino acid side chains of phenylalanine, tyrosine and tryptophan and giving CD spectra which is characteristic for native compact folded protein structure [29]. There was a gradual shift from 323 nm to 352 nm λ_em_ on increasing the concentration of GdHCl from 1 to 6 M, however shift was prominent only when the concentration of GdHCl were increased more than 3 M. Similar has been reported in case of hyperthermophilic asparaginase produced from the mutated *Pyroccocus furiosus* by Bansal, *et al* in 2011 [17]. The sequence of the first 15 N-terminal amino acids showed a wide variation with the known and reported protein sequence of L-asparaginase, however the conserved sequence of ATGGT was recorded [16] thus hence this L-asparaginase protein is new one which might attribute to its robust properties and low glutaminase activity. Purified L-asparaginase from *Bacillus licheniformis* RAM-8 was found to be highly effective against the cancer cell lines, Jurkat clone E6-1, MCF-7 and K-562 where IC_50_ was recorded in the range of less than 1 IU/ml. However the commercial L-asparaginase from *Erwinia* has been reported to have IC_50_ of 7.5 to 10.0 IU/ml and that of *E. coli* has IC_50_ of 1.0 IU/ml on the similar cell lines [30]. In this regards this, L-asparaginase can be considered as more potent and effective weapon in chemotherapy of tumors as compared to the present commercial preparations. Also this L-asparaginase was non-toxic against normal CHO cell line and human erythrocytes. Approximately 15–20% of patients treated with *E. coli* –derived asparaginase develop hypersensitivity and toxicity to the drug [31] hence this L-asparaginase could potentially be a better candidate under physiological conditions.

## Conclusions

L-asparaginase from *Bacillus licheniformis* RAM-8 was purified to apparent homogeneity and was found to be a homotetramer having molecular size of 134.8 kDa. This L-asparaginase is active and stable over wide range of temperature and pH and specific for it its natural substrate *i.e.* L-asparagine. Biophysical characterization concluded it to be robust structure of α/β mixed protein. The N-terminal sequence of this protein did not match with the known and reported L-asparaginase sequence hence protein is a new one. This L-asparaginase showed anticancerous effect against Jurkat clone E6-1, K-562 and MCF-7 cell lines and non-toxic effect against the heparinized blood or CHO test cell line.

## References

[1] Ohnuma T, Holland JF, Freeman A, Sinks LF (1970) Biochemical and harmacological studies with asparaginase in man. Cancer Res 30(9): 297–305. 4920133

[2] Wriston(Jr) JC, Yellin TO (1973) L-asparaginase: A Review. Adv. Enzym. 39: 185–248. 10.1002/9780470122846.ch3 4583638

[3] Lubkowski J, Palm GJ, Gilliland GL, Derst C, Rohm KH, et al (1996) Refined crystal structure of Acinetobacter glutaminasificans glutaminase-asparaginase. Eur. J. Biochem 241: 201–207. 10.1111/j.1432-1033.1996.0201t.x 8898907

[4] Neuman RE, McCoy TA (1956) Dual requirement of Walker carcinosarcoma 256 in vitro for asparagine and glutamine. Science. 124(3212): 124–125. 10.1126/science.124.3212.124 13337363

[5] Mashburn LT, Wriston JC Jr (1964) Tumor Inhibitory Effect of L-Asparaginase from Escherichia Coli. Arch Biochem Biophys 105: 450–452. 14186753 10.1016/0003-9861(64)90032-3

[6] Godfrin Y, Bertrand Y (2006) L-asparaginase Introduced into Erythrocytes for the Treatment of Leukaemia (ALL). BioMedES 1(1): 10–13.

[7] Broome JD, Schwartz JH (1967) Differences in the production of L-asparagine in asparaginase-sensitive and resistant lymphoma cells. Biochim Biophys Acta 138(3): 637–639. 6036864 10.1016/0005-2787(67)90569-2

[8] Prager MD, Bachynsky N (1968) Asparagine synthetase in asparaginase resistant and susceptible mouse lymphomas. Biochem Biophys Res Commun. 31(1): 43–47. 10.1016/0006-291x(68)90028-4 4869945

[9] Klumper E, Pieters R, Veerman AJ, Huismans DR, Loonen AH, et al (1995) In vitro cellular drug resistance in children with elapsed/refractory acute lymphoblastic leukemia. Blood. 86(10): 3861–3868. 7579354

[10] Duval M, Suciu S, Ferster A, Rialland X, Nelken B, et al (2002) Comparison of *Escherichia coli*–asparaginase with Erwinia-asparaginase in the treatment of childhood lymphoid malignancies: results of a randomized European Organization for Research and Treatment of Cancer–Children’s Leukemia Group phase 3 trial. Blood 99(8): 2734–2739. 11929760 10.1182/blood.v99.8.2734

[11] Surolia R, Pachauri M, Ghosh PC (2012) Preparation and characterization of monensin loaded PLGA nanoparticles: in vitro anti-malarial activity against *Plasmodium falciparum*. J Biomed Nanotechnol. 8(1): 172–81. 10.1166/jbn.2012.1366 22515105

[12] Mahajan RV, Saran S, Kameswaran K, Kumar V, Saxena RK (2012) Efficient production of L-asparaginase from *Bacillus licheniformis* with low-glutaminase activity: optimization, scale up and acrylamide degradation studies. Bioresour Technol. 125: 11–16. 10.1016/j.biortech.2012.08.086 23018158

[13] Shifrin S, Parrott CL, Luborsky SW (1974) In vitro assembly of L-asparaginase subunits. J Biol Chem 249: 1335–1340. 4604589

[14] Bradford MM (1976) Rapid and sensitive method for the quantitation of microgram quantities of protein utilizing the principle of protein-dye binding. Anal. Biochem. 72: 248–254. 10.1016/0003-2697(76)90527-3 942051

[15] Whitaker JR (1963) Determination of Molecular Weights of Proteins by Gel Filtration of Sephadex. Anal. Chem. 35(12): 1950–1953.

[16] Kumar S, Dasu VV, Pakshirajan K (2011) Purification and characterization of glutaminase-free L-asparaginase from Pectobacterium carotovorum MTCC 1428 Biores Tech. 102: 2077–2082. 10.1016/j.biortech.2010.07.114 20832300

[17] Bansal S, Srivastava A, Mukherjee G, Pandey R, Verma AK, et al (2012) Hyperthermophilic asparaginase mutants with enhanced substrate affinity and antineoplastic activity: structural insights on their mechanism of action FASEB J. 26(3): 1161–1171. 10.1096/fj.11-191254 22166247

[18] Lubran MM (1989) Hematologic side effects of drugs. Ann Clin Lab Sci. 19(2): 114–121. 2665627

[19] Aghaiypour K, Wlodawer A, Lubkowski J (2001) Do bacterial L-asparaginases utilize a catalytic triad Thr-Tyr-Glu? Biochim. Biophy. Acta. 1550: 117–128. 10.1016/s0167-4838(01)00270-9 11755201

[20] Aung HP, Bocola M, Schleper S, Röhm KH (2000) Dynamics of a mobile loop at the active site of Escherichia coli asparaginase. Biochem. Biophy. Acta. 1481: 349–359. 10.1016/s0167-4838(00)00179-5 11018727

[21] Prakasham RS, Hymavathi M, Rao CS, Arepalli SK, Rao JV, et al (2010) Evaluation of antineoplastic activity of extracellular asparaginase produced by isolated Bacillus circulans. Appl. Biochem. Biotechnol. 160: 72–80. 10.1007/s12010-009-8679-8 19543838

[22] Cammack KA, Marlborough DI, Miller DS (1972) Physical Properties and Subunit Structure of L-Asparaginase Isolated from Erwinia carotovora Biochem. J. 126: 361–379. 10.1042/bj1260361 PMC1178385 4561025

[23] Muller HJ, Boos J (1998) Use of L-asparaginase in childhood ALL. Crit. Rev. Oncol./Hematol. 28: 97–113. 10.1016/s1040-8428(98)00015-8 9768345

[24] Warangkar SC, Khobragade CN (2010) Purification, characterization, and effect of thiol compounds on activity of the Erwinia carotovora L-asparaginase. Enzyme Res. 2010: 1–10. 10.4061/2010/165878 PMC2956972 21048860

[25] Nagarethinam S, Nagappa AN, Udupa N, Rao VJ, Vanathi MB (2012) Microbial L-Asparaginase and its future prospects. Asian J Med Res. 1(4): 159–168.

[26] Leung-Toung R, Li W, Tam TF, Karimian K (2002) Thiol-dependent enzymes and their inhibitors: a review. Curr Med Chem. 9(9): 979–1002. 10.2174/0929867024606704 11966457

[27] Peterson RE, Ciegler A (1969) L-asparaginase production by Erwinia aroideae Appl. and Env. Microbiol. 18(1): 64. 10.1128/am.18.1.64-67.1969 PMC377887 5803630

[28] Willis RC, Woolfolk CA (1974) Asparagine utilization in Escherichia coli. J. Bacteriol. 118: 231–241. 10.1128/jb.118.1.231-241.1974 PMC246662 4595199

[29] Sharon MK, Nicholas CP (2000) The use of circular dichroism in the investigation of protein structure and function. Curr Protein Pept Sci 1, 349–384. 10.2174/1389203003381315 12369905

[30] Abakumova OY, Podobed OV, Karalkin PA, Kondakova LI, Sokolov NN (2012) Antitumor Activity of L-Asparaginase from Erwinia carotovora against Different Human and Animal Leukemic and Solid Tumor Cell Lines. Biochem (Mosc) Suppl Series B: Biomed Chem. 6(4): 307–316. 10.18097/pbmc20135905498 24479339

[31] Dhanam JG, Kannan S (2013) L-asparaginase- Types, Perspectives and Applications. Advanced BioTech. 13(1): 01–05.

